# Severe Renal Hemorrhage in a Pregnant Woman Complicated with Antiphospholipid Syndrome: A Case Report

**DOI:** 10.1155/2011/791094

**Published:** 2011-02-14

**Authors:** Shohei Kawaguchi, Kouji Izumi, Takahiro Nohara, Tohru Miyagi, Hiroyuki Konaka, Atsushi Mizokami, Eitetsu Koh, Mikio Namiki

**Affiliations:** Department of Integrative Cancer Therapy and Urology, Kanazawa University Graduate School of Medical Science, 13-1 Takara-machi, Kanazawa, Ishikawa 920-8641, Japan

## Abstract

Antiphospholipid syndrome is a systemic autoimmune disease with thrombotic tendency. Consensus guidelines for pregnancy with antiphospholipid syndrome recommend low-dose aspirin combined with unfractionated or low-molecular-weight heparin because antiphospholipid syndrome causes habitual abortion. We report a 36-year-old pregnant woman diagnosed with antiphospholipid syndrome receiving anticoagulation treatment. The patient developed left abdominal pain and gross hematuria at week 20 of pregnancy. An initial diagnosis of left ureteral calculus was made. Subsequently abdominal-pelvic computed tomography was required for diagnosis because of the appearance of severe contralateral pain. Computed tomography revealed serious renal hemorrhage, and ureteral stent placement and pain control by patient-controlled analgesia were required. After treatment, continuance of pregnancy was possible and vaginal delivery was performed safely. This is the first case report of serious renal hemorrhage in a pregnant woman with antiphospholipid syndrome receiving anticoagulation treatment and is an instructive case for urological and obstetrical practitioners.

## 1. Introduction

Antiphospholipid syndrome (APS) is a systemic autoimmune disease characterized by the presence of venous or arterial thromboembolism and/or pregnancy morbidity in association with antiphospholipid antibody (aPL). Consensus guidelines for cases of pregnancy with APS without a history of thrombosis recommend administration of low-dose aspirin in combination with unfractionated or low-molecular-weight heparin [1] because APS causes habitual abortion, intrauterine growth retardation, and severe toxemia of pregnancy at high rates. On the other hand, renal hemorrhage as an initial symptom of adverse events in administration of antiplatelet and anticoagulant drugs is very rare. Accordingly, it is difficult to assume that antiplatelet or anticoagulant drugs cause gross hematuria with severe flank pain. Here, we report a pregnant woman with APS who had severe renal hemorrhage during anticoagulation treatment.

## 2. Case Report

**Figure 1:**
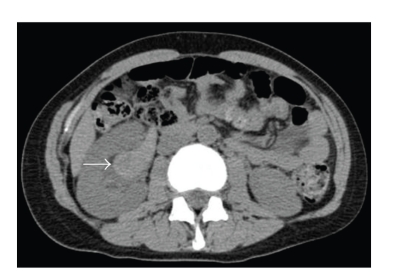
Axial CT image indicating that the right renal pelvis was filled with hematoma (arrow).

**Figure 2:**
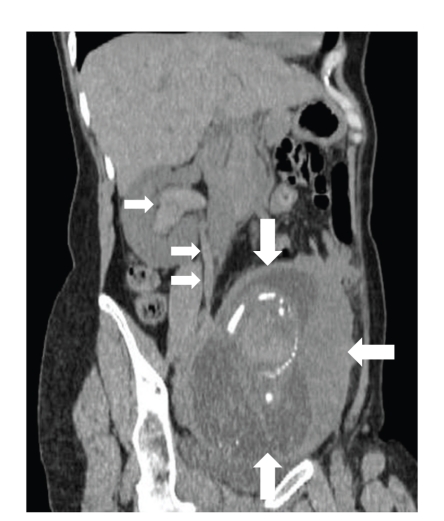
Longitudinal CT image indicating hematoma extending to the lower portion of the right ureter (small arrows) and a fetus in the uterus (large arrows).

A 36-year-old pregnant woman who had previously been diagnosed with APS had received anticoagulation treatment with 100 mg/day of aspirin and 12000 U/day of unfractionated heparin and had experienced abortion twice. She presented with gross hematuria at 20 weeks of pregnancy and discontinued aspirin administration. However, hematuria did not resolve, and lower left abdominal pain developed. Although hydronephrosis was not detected by ultrasound, left ureteral calculus was suspected from the patient's clinical course. Seven days later, hematuria and left abdominal pain developed again. The patient was treated conservatively with analgesics because ultrasound and plain abdominal X-ray results were unremarkable. Ten days later, the patient attended the hospital emergency department and was hospitalized urgently in urological ward for workup and treatment because of severe right back pain and severe gross hematuria developed. On admission, complete blood cell count (CBC) revealed elevated white blood cell counts (WBC 16.29 × 10^3^/*μ*L), decreased red blood cell and hemoglobin levels (RBC 2.76 × 10^6^/*μ*L and Hb 8.1 g/dL), and normal platelet counts (21.9 × 10^4^/*μ*L). The results of blood biochemical and clotting tests were unremarkable. There were no findings in physical examination except for a knocking pain of right costovertebral angle. Under an obstetrician's instruction, plain abdominal-pelvic computed tomography (CT) with reduced dose was performed although the patient was in second trimester. CT findings showed expanded right upper urinary tract and the hematoma in the right renal pelvis was suspected (Figures 1 and 2). There was no calculus and no mass lesion, and perinephric bleeding was not seen. The cause of bleeding in the right renal pelvis was unknown. Administration of unfractionated heparin was immediately discontinued. On day 2 after admission, severe back pain persisted and WBC was still elevated (26.64 × 10^3^/*μ*L). Therefore, antibiotic treatment was started for suspected urinary tract infection. On day 3, patient-controlled analgesia (PCA) with fentanyl was started for persistent pain by the anesthesiologist. Blood transfusion was performed on day 4 because CBC showed progression of anemia (Hb 6.7 g/dL). Ureteral stent placement under ultrasound guidance was performed because expansion of the right renal pelvis due to hematoma persisted. The pain and hematuria were subsequently relieved. On day 16, administration of unfractionated heparin was resumed based on consultation with an obstetrician and a hematologist. The patient was discharged on day 21. Although outpatient anticoagulation treatment was continued, gross hematuria and back pain did not recur. At 36 weeks of pregnancy, the patient was admitted to hospital and continuous instillation of heparin was started. One week later, she gave birth to a female infant with a birth weight of 2190 g by vaginal delivery after discontinuation of heparin infusion. Apgar scores at 1 and 5 min were 9 and 10, respectively. Continuous instillation of low-molecular-weight heparin was started postpartum. Abdominal-pelvic CT and urine cytology performed on day 4 postpartum revealed no abnormalities in the urinary tract. Heparin infusion was then switched to oral low-dose aspirin. The ureteral stent was withdrawn on day 8, and the patient has not developed any symptoms, such as pain or hematuria.

## 3. Discussion

APS is a syndrome first described by Hughes et al. as the anticardiolipin syndrome in 1983 [2] and is a multisystem disease characterized by venous or arterial thrombosis and/or pregnancy morbidity in association with aPL [3]. APS causes widespread arterial and venous thrombosis, including stroke, myocardial infarction, and deep-vein thrombosis. Pregnancy morbidity with APS is not necessarily complicated by thrombosis, and habitual abortion or toxemia usually develops as unique symptom. Aspirin and/or heparin are usually administered as anticoagulation treatment during pregnancy. In contrast, some cases show development of hemorrhagic tendency, which is considered to be due to thrombocytopenia by platelet destruction occurring with aPL binding. An in vitro study also indicated that lupus anticoagulant inhibits adhesion and aggregation of platelets, and it is possible that aPL binds to platelets and reduces platelet function [4]. In this case, because thrombocytopenia or abnormality of clotting test was not detected throughout the clinical course, anticoagulation treatment was considered to be the cause of renal bleeding.

Several hemorrhagic complications have been reported, such as subdural hematoma, ovarian hemorrhage, and intestinal hemorrhage as the adverse events of anticoagulation treatment [5]. There have been a few reports of genitourinary symptoms, such as pericapsular renal bleeding, adrenal hemorrhage, or hemorrhagic cystitis with secondary APS [6, 7]. Various renal complications, including renal artery or venous thrombosis, renal infarct, hypertension, renal failure, and various glomerular lesions, have been reported as APS nephropathy [8]. However, to our knowledge, there have been no previous reports of severe renal hemorrhage with APS, and the present case was considered very rare.

With regard to adverse effects of radiation on the fetus, it is unlikely that single pelvic CT would have teratogenic effects. However, the relative risk of carcinogenesis by radiation exposure was reported to be 1.29 in the second trimester (3.19 and 1.30 in first and third trimesters, resp.) [9]. Therefore, radiation exposure during pregnancy should be avoided as much as possible. The patient had symptoms on both sides, and the symptoms worsened rapidly. Abdominal-pelvic CT was performed because it was difficult to determine her clinical condition based only on ultrasound findings. Furthermore, various renal morbidities should be considered in patients with APS. Although the patient described here was in the second trimester and the potential effect on the fetus was less than in the first trimester, radiodiagnosis during pregnancy should always require careful consideration.

We reported a case of renal hemorrhage in a pregnant woman with APS. This is the first case report of a pregnant woman with APS developing serious renal hemorrhage. The symptoms were relieved with ureteral stent and PCA, and successful delivery was achieved. Administration of aspirin and heparin is often performed in cases of pregnancy with APS, and it is necessary to pay attention to the hemorrhagic symptoms. In addition, diagnosis should be made as soon as possible the symptoms emerged. However, it is difficult to carry close examinations such as contrast-enhanced CT, magnetic resonance image, or angiography because the patient is under a complicated condition with APS and pregnancy. Close cooperation with related specialists should be performed in examination and treatment of such cases. Although to repeat examination using ultrasound is thought as a principle method to quest the cause of severe back pain and hematuria in pregnant patients with APS, it may be needed to perform CT speedily with obstetrician's instruction in case the cause of symptoms is unclear. This is an instructive case for urological and obstetrical practitioners.
